# Matt: Local Flexibility Aids Protein Multiple Structure Alignment

**DOI:** 10.1371/journal.pcbi.0040010

**Published:** 2008-01-11

**Authors:** Matthew Menke, Bonnie Berger, Lenore Cowen

**Affiliations:** 1 Computer Science and Artificial Intelligence Laboratory, Massachusetts Institute of Technology, Cambridge, Massachusetts, United States of America; 2 Department of Mathematics, Massachusetts Institute of Technology, Cambridge, Massachusetts, United States of America; 3 Department of Computer Science, Tufts University, Medford, Massachusetts, United States of America; Fox Chase Cancer Center, United States of America

## Abstract

Even when there is agreement on what measure a protein multiple structure alignment should be optimizing, finding the optimal alignment is computationally prohibitive. One approach used by many previous methods is aligned fragment pair chaining, where short structural fragments from all the proteins are aligned against each other optimally, and the final alignment chains these together in geometrically consistent ways. Ye and Godzik have recently suggested that adding geometric flexibility may help better model protein structures in a variety of contexts. We introduce the program Matt (Multiple Alignment with Translations and Twists), an aligned fragment pair chaining algorithm that, in intermediate steps, allows local flexibility between fragments: small translations and rotations are temporarily allowed to bring sets of aligned fragments closer, even if they are physically impossible under rigid body transformations. After a dynamic programming assembly guided by these “bent” alignments, geometric consistency is restored in the final step before the alignment is output. Matt is tested against other recent multiple protein structure alignment programs on the popular Homstrad and SABmark benchmark datasets. Matt's global performance is competitive with the other programs on Homstrad, but outperforms the other programs on SABmark, a benchmark of multiple structure alignments of proteins with more distant homology. On both datasets, Matt demonstrates an ability to better align the ends of α-helices and β-strands, an important characteristic of any structure alignment program intended to help construct a structural template library for threading approaches to the inverse protein-folding problem. The related question of whether Matt alignments can be used to distinguish distantly homologous structure pairs from pairs of proteins that are not homologous is also considered. For this purpose, a *p*-value score based on the length of the common core and average root mean squared deviation (RMSD) of Matt alignments is shown to largely separate decoys from homologous protein structures in the SABmark benchmark dataset. We postulate that Matt's strong performance comes from its ability to model proteins in different conformational states and, perhaps even more important, its ability to model backbone distortions in more distantly related proteins.

## Author Summary

Proteins fold into complicated highly asymmetrical 3-D shapes. When a protein is found to fold in a shape that is sufficiently similar to other proteins whose functional roles are known, this can significantly aid in predicting function in the new protein. In addition, the areas where structure is highly conserved in a set of such similar proteins may indicate functional or structural importance of the conserved region. Given a set of protein structures, the protein structural alignment problem is to determine the superimposition of the backbones of these protein structures that places as much of the structures as possible into close spatial alignment. We introduce an algorithm that allows local flexibility in the structures when it brings them into closer alignment. The algorithm performs as well as its competitors when the structures to be aligned are highly similar, and outperforms them by a larger and larger margin as similarity decreases. In addition, for the related classification problem that asks if the degree of structural similarity between two proteins implies if they likely evolved from a common ancestor, a scoring function assesses, based on the best alignment generated for each pair of protein structures, whether they should be declared sufficiently structurally similar or not. This score can be used to predict when two proteins have sufficiently similar shapes to likely share functional characteristics.

## Introduction

The problem of constructing accurate protein multiple structure alignments has been studied in computational biology almost as long as the better-known multiple sequence alignment problem [1]. The main goal for both problems is to provide an alignment of residue–residue correspondences in order to identify homologous residues. When applied to closely related proteins, sequence-based and structure-based alignments typically give consistent answers even though most sequence alignment methods are measuring statistical models of amino acid substitution rates, whereas most structure-based methods are seeking to superimpose C-alpha atoms from corresponding backbone 3-D coordinates while minimizing geometric distance. However, as has been known for some time [2], these answers can diverge when aligning distantly related proteins; most relevant, it is still possible to find good structural alignments when sequence similarity has evolutionarily diverged into “the twilight zone” [3]. In the twilight zone, distantly related proteins can still share a *common core* structure containing regions, including conserved secondary-structure elements and binding sites, in which the chain retains the topology of its folding pattern (see [4] for a recent survey). Structural information can align the residues in this common core, even after the sequences have diverged too far for successful sequence-based alignment, because structural similarity is typically more evolutionarily conserved [5–7]. (While divergent sequence with conserved structure is the typical case and the one that structural alignment algorithms that respect backbone order such as Matt seek to handle, there are also well-known examples where structure has diverged more rapidly than sequence; see, for example, Kinch and Grishin [8] and Grishin [9].)

Applications of multiple structure alignment programs include understanding evolutionary conservation and divergence, functional prediction through the identification of structurally conserved *active sites* in homologous proteins [10], construction of benchmark datasets on which to test multiple sequence alignment programs [6], and automatic construction of profiles and threading templates for protein structure prediction [4,11]. It has also recently been suggested that multiple structure alignment algorithms may soon become an important component in the best multiple sequence alignment programs. As more protein structures are solved, there is an ever-increasing chance that a given set of sequences to be aligned will contain a subset with structural information available. To date, however, only a handful of multiple sequence alignment programs are set up to take advantage of any available structural data [6,12].

Pairwise structure alignment programs fall into three broad classes: the first class, to which Matt belongs, are aligned fragment pair (AFP) chaining methods [13,14] which do an all-against-all best transformation of short protein fragments from one protein structure onto another, and assemble these in a geometrically consistent fashion. The second class (which includes the popular Dali [15]), look at pairwise distances or contacts *within* each structure separately, then try to find a maximum set of corresponding residues that obey the same distance or contact relationships in pairs of structures—these are called *distance matrix* or *contact map* methods. The third class consists of everything else, from geometric hashing [16] borrowed from computer vision to an abstraction of the problem to secondary structural elements [17]. Some protein structure alignment programs are *nonsequential;* that is, they allow residue alignments that are inconsistent with the linear progression of the protein sequence [18–21]. Most enforce an alignment consistent with the sequential ordering of the residues along the backbone—Matt belongs to the class of sequential protein aligners. There are strengths to both approaches: most useful protein alignments are sequential; however, nonsequential protein aligners can handle cases where there is a reordering of domains, and circular permutations [9].

Multiple structure alignment programs are typically built on top of pairwise structural alignment programs. Even simplified variants of structure alignment are known to be NP-hard [22,23]; important progress has been recently been made in theoretically rigorous approximation guarantees [24] for pairwise structural alignment using a class of single optimality criteria scores such as the Structal score [1], and also in provably fast parameterized algorithms for the pairwise structural alignment problem in the nonsequential case [20].

### Performance Metrics

There are two related problems that protein structure alignment programs are designed to address. The first we will call the *alignment* problem, where the input is a set of *k* proteins that have a conserved structural common core, where the common core is defined as in Eidhammer et al. [25] as a set of residues that can be simultaneously superimposed with small structural variation. The desired output consists of a superimposition of the proteins in 3-D space, coupled with the list of which amino acid residues are declared to be in alignment and part of the core. The second problem, which we will call the *discrimination* problem, takes as input a pair of protein structures, and is supposed to output a yes/no answer (together with an associated score or confidence value) as to whether a good alignment can be found for these two protein structures or not. We discuss how to measure performance on the alignment problem first, and then on the discrimination problem below.

The classical geometric way to measure the quality of a protein structural alignment involves two parameters: the number of amino acid residue positions that are found to participate in the alignment (and are therefore found to be part of the conserved structural core), as well as the average pairwise root mean squared deviation (RMSD) (where RMSD is calculated from the best rigid body transformation using least squares minimization [26]) between backbone atoms placed in alignment in the conserved core. Clearly, this is a bi-criteria optimization problem: the goal is to *minimize* the RMSD of the conserved core while *maximizing* the number of residues placed in the conserved core.

We first take a traditional geometric approach: reporting for all programs and all benchmark datasets, the average number of residues placed into the common core structure, alongside the average RMS of the pairwise RMSDs among all pairs of residues that participate in a multiple alignment of a set of structures. In addition, results are compared against Homstrad reference alignments. This approach follows Godzik and Ye's evaluation of their multiple structure alignment program, POSA [27].

More sophisticated geometric scoring measures have also been suggested, some to collapse the bi-criteria optimization problem into a single score to be optimized [28], such as the Structal score [1], others to incorporate more environmental information into the similarity measure, such as secondary structure, or solvent accessibility [29,30]. The *p*-value score that we develop to handle the discrimination problem, described below, is a collapse of the bi-criteria optimization problem into one score that provides a single lens on pairwise alignment quality.

An alternative approach to measuring the performance of a structure alignment algorithm comes from the discrimination problem directly. Here, the measure is typically ROC curves; looking for the ratio of true and false positives and negatives from a “gold-standard” classification for what is alignable or not, based either on decoy structures or a classification scheme such as SCOP or CATH. Indeed, a possible concern with adding flexibility to protein structure would be that the added flexibility in our alignment might lead to an inability to distinguish structures that should have good alignments from those that do not. We therefore test our ability to distinguish true alignable structures from decoys on the SABmark dataset (which comes with a ready-made set of decoy structures) as compared to competitor programs.

### Our Contribution

We introduce the program Matt (Multiple Alignment with Translations and Twists), an AFP fragment chaining method for pairwise and multiple sequence alignment that outperforms existing popular multiple structure alignment methods when tested on standard benchmark datasets. At the heart of Matt is a relaxation of the traditional rigid protein backbone transformations for protein superimposition, which allows protein structures *flexibility* to bend or rotate in order to come into alignment. While flexibility has been introduced into the study of protein folding in the context of docking [31,32], particularly for the modeling of ligand binding [33], and more recently in decoy construction for ab initio folding algorithms [34,35], it has only recently been incorporated into general-purpose pairwise [14,36] and multiple [27] structure alignment programs. There are two reasons it makes sense to introduce flexibility into protein structure alignment. The first is the main reason that has been addressed in previous work, namely, aligning proteins that do not align well by means of rigid body transformations because their structures have been determined in different conformational states: a well-known example is that the fold of a protein will change depending on whether it is bound to a ligand or not [33]. Matt is designed to also address the second reason to introduce flexibility into protein structure alignment, namely to handle structural distortions as we align proteins whose shape becomes increasingly divergent outside the conserved core.

We find that at each fixed value for number of aligned residues, Matt is competitive with other recent multiple structure alignment programs in average RMSD on the popular Homstrad [37] and outperforms them on the SABmark [38] benchmark datasets (see Tables 1, 2, and 3). We emphasize again that this is an *apples-to-apples* comparison of the best (i.e., the standard least squares RMSD minimization) rigid body transformation for Matt's alignments, just as it is for the other programs—while Matt allows impossible bends and breaks in intermediate stages of the algorithm, it is stressed that the final Matt alignments and RMSD scores come from legal, allowable “unbent” rigid body transformations. We also present RMSD/alignment length tradeoffs for Matt performance on the same datasets. In the case of Homstrad, where a manually curated “correct” structural alignment is made available as part of the benchmark, Matt alignments are also measured against the reference alignments, where we are again competitive with or outperforming previous structure alignment programs (see Table 1).

**Table 1:**
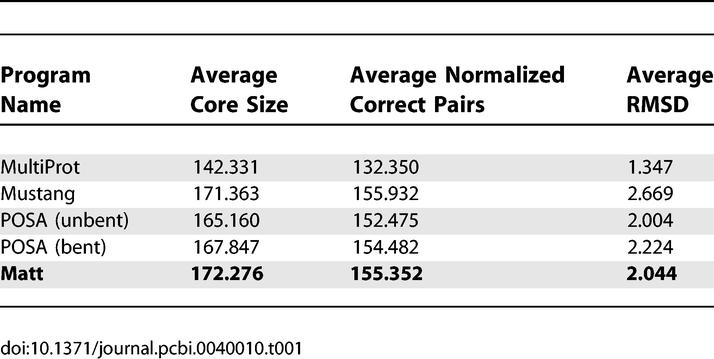
Homstrad Performance Comparison

**Table 2:**
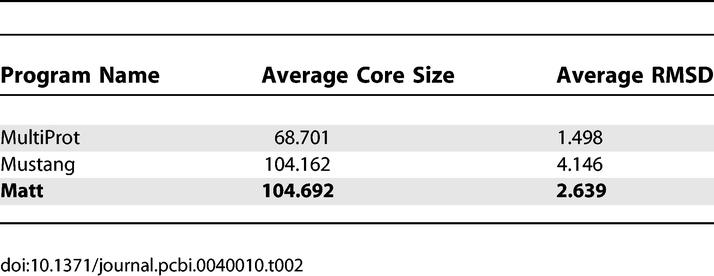
SABmark Superfamily Performance Comparison

**Table 3:**
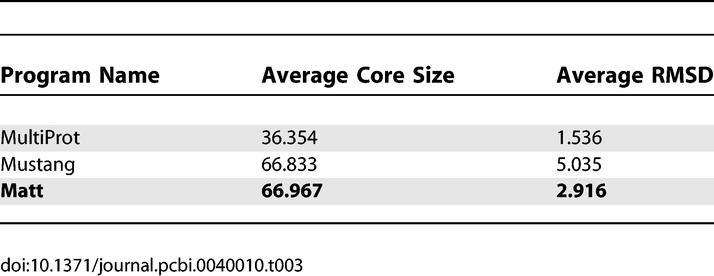
SABmark Twilight Zone Performance Comparison

In addition, Matt's ability to distinguish truly alignable folds from decoy folds is tested with the standard benchmark SABmark set of alignments and decoys [38]. The SABmark decoy set was constructed to contain, for each alignable subset, decoy structures that belong to a different SCOP superfamily, but whose sequences align reasonably well according to BLAST [38]. Thus, they may be more similar at the local level to the true positive examples, and thus fool a structure alignment program better than a random structure. Here, we tested both the “unbent” Matt alignments described above, but also the “bent” Matt alignments, where the residues are aligned allowing the impossible bends and breaks. We test Matt's performance both against the decoy set and also against random structures taken from the Protein Data Bank (PDB; http://www.rcsb.org/pdb). We use Matt's performance on the truly random structures to generate a *p*-value score for pairwise Matt alignments. Rather than choose from among the large number of competitor pairwise structural alignment programs, Matt was instead tested against other *multiple* structure aligners, in fact the same programs we used for measuring how well they aligned protein families known to have good alignments. The exception was that we also tested the FlexProt program [36], a purely pairwise structure alignment program that was of special interest because it also claims to handle flexibility in protein structures.

We have made Matt along with its structural alignments available at http://groups.csail.mit.edu/cb/matt and http://matt.cs.tufts.edu so anyone can additionally compute any alternate alignment quality scores they favor.

### Related Work

The only general protein structure alignment programs that previously tried to model flexibility are FlexProt [36] and Ye and Godzik's Fatcat [14] (both for pairwise alignment), and Fatcat's generalization to multiple structure alignment, POSA [27]. Fatcat is also an AFP chaining algorithm, except it allows a globally minimized number of translations or bends in the structure if it improves the overall alignment. In this way, it is able to capture homologous proteins with hinges, or other discrete points of flexibility, due to conformational change. Our program Matt is fundamentally different: it instead allows flexibilities *everywhere* between short fragments—that is, it does not seek to globally minimize the number of bends, but rather allows continuous small local perturbations in order to better match the “bent” RMSD between structures. Because Matt allows these flexibilities, it can put strict tolerance limits on “bent” RMSD, so it only keeps fragments that locally have very tight alignments. Up until the last step, Matt allows the dynamic program to assemble fragments in ways that are structurally impossible—one chain may have to break or rotate beyond the physical constraints imposed by the backbone molecules in order to simultaneously fit the best transformation. This is repaired in a final step, when the *residue to residue* alignment produced by this unrealistic “bent” transformation is retained; the best rigid-body transformation that preserves that alignment is found, and then either output along with the residue–residue correspondences produced by the “bent” Matt alignment *or* extended to include as yet unaligned residues that fall within a user-settable maximum RMSD cutoff under the new rigid-body transformation to form the final Matt “unbent” alignment.

### Matt Implementation

Matt accepts standard PDB files as input, and outputs alignment coordinates in PDB format as well. In addition, when only two structures are input, Matt outputs a *p*-value for whether or not Matt believes the structures are alignable (see below). There is an option to output the “bent” structures in PDB format. Matt also outputs the sequence alignment derived from the structural alignment in FASTA format and a Rasmol script to highlight aligned residues. Windows and Linux binaries and source code are available at http://groups.csail.mit.edu/cb/matt and http://matt.cs.tufts.edu.

### Algorithm Overview

The input to Matt is a set of *g* groups of already multiply aligned protein structures (at the beginning of the algorithm, each structure is placed by itself into its own group). The iterative portion of the Matt algorithm runs *g* − 1 times, each time reducing the number of separate groups by 1 as it merges two sets of aligned structures in a progressive alignment. As we discuss in detail below, the Matt alignments produced in the iterative portion are not geometrically realized by rigid body transformations: they allow local “bends” in the form of transpositions and rotations. Once there is only one group left, Matt enters a final pass, where it corrects the global alignment into an alignment that obeys a user-settable RMSD cutoff by means of geometrically realizably rigid-body transformations. A flowchart describing the stages of the Matt algorithm appears in Figure 1.

**Figure 1:**
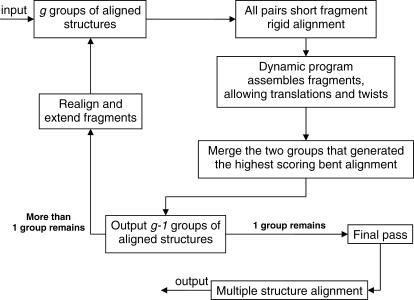
Overview of the Matt Algorithm

#### The iterative portion: fragment pairs.

There are three main phases to the iterative portion of the Matt algorithm. The first phase is similar to what is done by many existing AFP chaining residues: for simplicity, it is first described here for pairwise alignment (that is, when each group consists of a single structure). Matt considers fragments of five to nine adjacent amino acid residues. A fragment pair is a pair of fragments of equal length, one from each structure. For every fragment pair, between any pair of structures, an alignment score is calculated based on an estimated *p*-value of the minimum RMSD achievable by a rigid-body transformation of the C-alpha atoms of one fragment onto the other. *p*-values are estimated by generating a table of random RMSD alignments of the National Center for Biotechnology Information (NCBI) nonredundant PDB.

The generalization from aligning fragments from two structures to aligning fragments from two groups of multiple structure alignments is straightforward. The alignment score of a pair of fragments, one from each group alignment, is calculated based on a single rigid-body transformation acting on all of a group's structures together.

#### Dynamic programming assembly with translations and twists.

Matt's main novel contribution involves how we assemble these short fragments into a global alignment. Note that Matt is an alignment program that respects the sequential order of residues along the backbone, so it only assembles aligned fragments that are consistently ordered.

Matt iteratively builds up longer and longer sets of aligned fragments using dynamic programming. When deciding whether to chain two sets of aligned fragments together, Matt uses a score based on the sum of the alignment scores of the individual aligned fragments together with a penalty based on the geometric consistency of the transformations associated with deforming the backbone of one set onto the other. Transformation consistency cutoffs were determined empirically using a fifth of the Homstrad benchmark dataset. The consistency score (specified exactly in Methods below) is a function of both relative translation and relative rotation angles; the angles are calculated using quaternions for speed.

Matt finds the highest-scoring assembly for all pairs of groups of aligned structures that were input. It then chooses the pair of groups with the highest-scoring assembly, and uses that assembly to create a new multiple alignment that merges those two groups. If only one group remains, the algorithm proceeds to the final pass; otherwise, it enters the realign and extend phase before looping back to calculate all fragment pairs again.

#### Realign and extend phase.

The realignment phase does not change the residue correspondences in the multiple alignment, but tries to find local transformations that tighten RMSD in the aligned fragments in the newly merged group. It is described in more detail in Methods.

The extension phase is then called. The multiple alignment is extended greedily off both ends of all its fragments as long as average RMSD is below a cutoff (4 Å). Extended fragments are allowed to overlap for up to five residues in this phase (if this produces any inconsistencies in the alignment, note that they are fixed in the next dynamic programming iteration). When only one group of structures remains, the result is the *bent* Matt alignment. The algorithm enters the final pass to produce the *rigid bent* and *unbent* Matt alignments.

#### Final pass.

The input to the final pass is simply which residues have been aligned with which in the final bent alignment; that is, the multiple sequence alignment derived by the bent multiple structure alignment Matt has generated. Once the mapping of *which* residues are to be aligned has been fixed, finding the associate transformation that optimizes RMSD of the aligned residues of one structure against a set of structures aligned to a reference structure is straightforward. We build up a single multiple structure alignment from such transformations using a similar method to that introduced by Barton and Sternberg [39]. In particular, additional protein structures are added progressively to the overall alignment. Each time a new structure is added, all existing structures are “popped out” in turn, and realigned back to the new reference alignment. This brings the atoms into tighter and tighter alignment.

The resulting rigid RMSD Matt alignment leaves the sequence alignment from the bent step *unchanged*, and thus only includes sets with five or more contiguous residues. This alignment is what we call the *rigid bent* Matt alignment, below. We then do a final pass to add back shorter segments. In particular, now that we have a final global multiple structure alignment, we greedily add back in fragments of four or fewer residues that fall between already aligned fragments but whose RMSD is below a user-settable cutoff. That user-settable cutoff is entirely responsible for the different tradeoffs between average RMSD and number of aligned residues that we obtain (see Figures 2 and 3)—in the comparisons with other programs, the cutoff was uniformly set at 5 Å.

**Figure 2:**
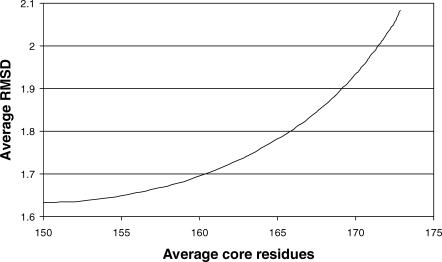
Matt Homstrad Performance Tradeoffs Average pairwise RMSD versus average number of residue positions placed in the common core for the Homstrad multiple alignment benchmark.

**Figure 3:**
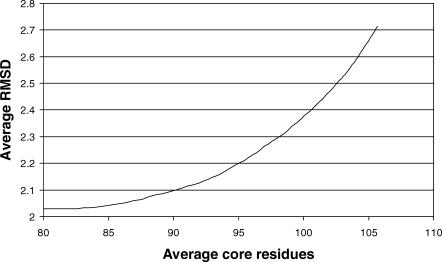
Matt SABmark Performance Tradeoffs Average pairwise RMSD versus average number of residue positions placed in the common core for the SABmark superfamily benchmark.

## Results

### The Benchmark Datasets

Perhaps the most popular dataset for testing protein structural alignment programs is Homstrad [37], which is a manually curated set of 1,028 alignments, each of which contains between two and 41 structures. Homstrad contains highly homologous proteins, with similarity comparable to the *family* level of the hierarchical SCOP [40] structural classification database. In this paper, in order to be comparable to the results for POSA presented in [27], we test only on the 399 Homstrad alignments with more than two structures in the alignment (that is, Homstrad sets with between three and 41 structures that necessitate a multiple rather than a pairwise structure alignment program).

We also test Matt on the superfamily and twilight zone SABmark [38] benchmark datasets. The superfamily set contains 3,645 domains sorted into 426 subsets representing structures at the superfamily level of the SCOP hierarchy, a set designed to be well-distributed in known protein space, and presumably containing more remote homologs than Homstrad. The twilight zone set contains 1,740 domains sorted into 209 subsets whose homology is even more remote than the superfamily set. Both the superfamily and twilight zone sets have subsets containing between three and 25 structures.

Since the “correct” alignments provided by SABmark are generated automatically from existing structure alignment programs, we do not report the percentage of “correctly” aligned residue pairs as we did for the manually curated Homstrad, but rather report only the objective geometric measures of alignment quality (number of residues placed in the conserved core, and average pairwise RMSD among residues placed in the combined core).

SABmark additionally provides a set of decoy structures for nearly all its 462 sets of alignable superfamily (and 209 alignable twilight zone) sets of structures. We constructed a *decoy* discrimination test suite as follows. Each SABmark superfamily (or twilight zone) test set comes with an equal number of decoy structures with high sequence similarity (see [38]). For each test set, a random pair of structures in the positive set (that belong to the same SCOP superfamily and are supposed to align) and a random decoy were selected. Then a *random* discrimination test suite was similarly constructed, the only difference being that the decoy was chosen to be a random structure in a different SABmark set, not a decoy structure that was specifically chosen to have high sequence similarity to the positive set.

### The Programs to Which Matt Is Compared

On Homstrad, we compare Matt to three recent multiple structure alignment programs: listed in alphabetical order, they are MultiProt [41], Mustang [42], and POSA [27]. Note that MultiProt has both sequential and nonsequential alignment options; we compare against the option that, like Matt, respects sequence order. MultiProt is an AFP program that uses rigid body superimposition. Mustang uses a combination of short fragment alignment, contact maps, and consensus-based methods. We were particularly eager to test Matt against POSA, because it is the only other multiple structure alignment program that allows flexibility, though as discussed in the Introduction, POSA's flexibility is more limited. POSA outputs two different structural alignments: one comes from the version of POSA that disallows bends, and the other from the version with limited bends allowed. We test both versions, and results appear in Table 1.

We were not able to obtain POSA code. (Our statistics on Homstrad come from POSA alignments provided by the authors as supplementary data). Because we were not able to obtain POSA code, we were not able to test POSA on all of SABmark, but we do compare Mustang and MultiProt to Matt on the entire SABmark benchmark. On the other hand, we were able to submit individual sets of SABmark structures to the POSA online server; POSA sometimes did nearly as well as Matt on the examples we tested, but other times, it missed finding alignable structures entirely. We show both cases in two in-depth examples: Figure 4 shows alignments of Matt, MultiProt, Mustang, and POSA on a seven-bladed β-propeller, and Figure 5 shows alignments of the four programs on a set of left-handed β-helix structures. POSA and Matt are the only algorithms that successfully align all seven blades of the β-propeller. POSA, however, entirely misses the alignable regions in the β-helix fold.

**Figure 4:**
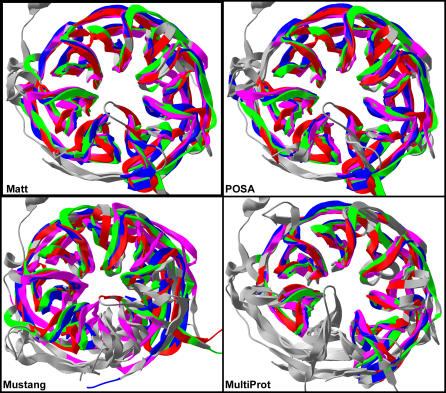
Comparative β-Propeller Alignments The four SABmark domains in the set Group 137, consisting of seven-bladed β-propellers as aligned by Posa, Mustang, MultiProt, and Matt. Backbone atoms that participate in the common core of the alignment show up colored as red (PDB ID d1nr0a1), green (PDB ID d1nr0a2), blue (PDB ID d1p22a2), and magenta (PDB ID d1tbga); residues in all four chains that are not placed into the alignment by the tested algorithm are shown in gray. These pictures were generated by the Swiss PDB Viewer (DeepView) [43].

**Figure 5:**
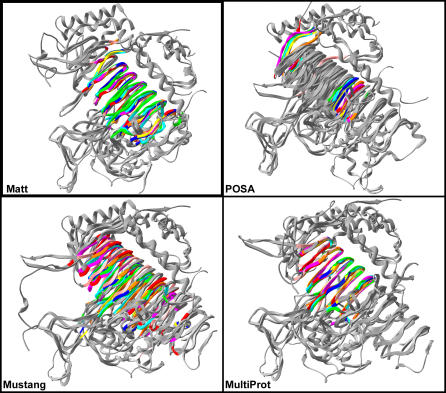
Comparative β-Helix Alignments Aligned portions of the eight SABmark domains in the set Group 144, consisting of the left-handed β-helix fold as aligned by Posa, Mustang, MultiProt, and Matt. Backbone atoms that participate in the common core of the alignment show up colored as red (PDB ID d1hm9a1), green (PDB ID d1kk6a), blue (PDB ID d1krra), magenta (PDB ID d1lxa), yellow (PDB Is that are not placed into the alignment by the tested algorithm are shown in gray. These pictureD d1ocxa), orange (PDB ID d1qrea), cyan (PDB ID d1xat), and pink (PDB ID d3tdt); residues in all three chains were generated by the Swiss PDB Viewer (DeepView) [43].

For the discrimination problem, Matt is compared against MultiProt and Mustang again, but also against FlexProt [36]. FlexProt has an option to allow from zero to four bends in the aligned structure, which is specified at runtime. FlexProt scores each of these structures by length of the alignment found. On each structure, the alignment with the number of bends that produces the highest-scoring alignment is output. In the case of both POSA and FlexProt, the “bent” alignment outputs the (best rigid-body transformation) RMSD of the aligned structures with bends allowed, and the “unbent,” or regular, version outputs the RMSD of the best rigid-body transformation that places the same set of residues in alignment as the bent version.

### Performance

Table 1 shows the following quantities for each program on the 399 Homstrad reference alignments that contain at least three structures each (this is the identical set of reference alignments on which POSA was tested). The first field is the average number of residues placed in the common core. The second is “average normalized correct pairs” computed according to the Homstrad reference alignments. This quantity is computed as follows: for each set of structures, we look at every pair of aligned residues that also participate in a Homstrad correct alignment. Then, we normalize based on the number of structures in the set (so the alignments in the set of 41 structures do not weight more heavily than the alignments in the set of three structures), dividing by the number of pairs of distinct structures in the reference set. (Note that, as discussed in [42], having additional pairs placed into alignment that Homstrad does not consider part of the “gold-standard” alignment is a positive, not a negative, if RMSD remains low. This is because declaring a pair of nearly aligned residues “aligned” or not is a judgment call that Homstrad makes partially based on older multiple structure alignment programs whose performance is weaker than the most recent programs.) The second column is the same “average RMSD” measure that POSA reports: the average RMS of the pairwise RMSDs among all pairs of residues that participate in a multiple alignment in a set of structures.

We downloaded and ran MultiProt and Mustang and computed RMSD statistics ourselves. POSA is not available for download, but is only accessible as a webserver; however, Homstrad alignments are available online at http://fatcat.burnham.org/POSA/POSAvsHOM.html. POSA's Web site provides two sets of multiple alignments: one derived from running their algorithm allowing geometrically impossible bends, and one running an unbent version of their algorithm. Note that for POSA's *bent* alignments, we had to recalculate RMSD from the multiple sequence alignment provided from their bent alignments, because unbent RMSD based on bent alignments was not provided on their Web site. It is of independent interest that, as expected, POSA's unbent version has better RMSD, while POSA's bent version finds more residues participating in the alignments overall.

Matt scores slightly better than POSA on Homstrad. Matt's average core size is comparable with that of Mustang, but Matt has a lower RMSD. The size of the alignments that MultiProt finds are much smaller than for the other programs (though its average RMSD is therefore much lower): this becomes even more pronounced on the more distant structures in SABmark (see Figure 3). Note that the core-size/RMSD tradeoff of Matt is very sensitive to cutoffs set in the last pass of the Matt algorithm, when it is decided what segments of less than five consecutive residues are added back into the alignment. Throughout this paper, the results reported in our tables come from setting the cutoff at 5 Å. By comparison, if the cutoff is set at 3.5 Å, Matt achieves 168.038 average core size, 153.362 average normalized pairs correct, and 1.862 average RMSD on Homstrad. Full tradeoff results on RMSD versus number of residues based on changing the last pass cutoff appear in Figure 2. Note that Matt's cutoffs for allowable bends were trained on a random 20% of the Homstrad dataset.

While Matt competes favorably with the other programs on Homstrad, Matt was designed for sets of more distantly related proteins than appear in the Homstrad benchmark. Thus, the best demonstration of the advantage of the Matt approach appears on the more distantly related proteins in the SABmark benchmark sets. Here, Matt is seen to do exactly what was hoped: by detouring through bent structures, it finds rigid RMSD alignments that place as many residues in the conserved alignment as Mustang does (and more than 50% more than MultiProt does) while reducing the average RMSD from that of Mustang by more than 1.4 Å (see Tables 2 and 3). It should again be emphasized that none of Matt's parameters were trained on SABmark.

Looking by hand through the alignments, MultiProt consistently aligns small subsets of residues correctly, but leaves large regions unaligned that both Mustang and Matt believe to be alignable. Mustang, on the other hand, frequently misaligns regions, particularly in the case when there are many α-helices tightly packed in the structure. On two of the twilight zone sets, Mustang fails to find anything in the common core. Altogether on the twilight zone set, there are four sets of structures for which Mustang fails to find at least three residues in the common core (and there is one set of structures in the superfamily set where Mustang also fails to find anything in the common core). Though the effect is negligible, these four sets are removed from Mustang's average RMSD calculation.

Although these tables show overall performance, it is also helpful to look at actual examples. We pulled two example reference sets out of the SABmark superfamily benchmark. Figure 4 shows the Matt alignment versus MultiProt, POSA, and Mustang alignments of the SABmark structures in the set labeled Group 137 (β-propellers; PDB IDs d1nr0a1, d1nr0a2, d1p22a2, and d1tbga). POSA does second best to Matt here, and in fact, the overall alignment of the structures in POSA is most similar to Matt—the same propeller blades are overlaid in both alignments. Although it is hard to see in the picture, Mustang is superimposing the wrong blades, which accounts for the terrible RMSD. MultiProt makes a similar error, but then gets a low RMSD by aligning less of the structure. Figure 5 shows a Matt alignment of the SABmark structures in the set labeled Group 144 (β-helices; PDB IDs d1hm9a1, d1kk6a, d1krra, d1lxa, d1ocxa, d1qrea, d1xat, and d3tdt). Here, POSA does very poorly, only finding a very small set of residues to align. MultiProt again aligns the portion that it declares in the common core very tightly (this is a theme throughout the SABmark dataset), but it only places five rungs in the common core. Both these figures were generated using the Swiss PDB Viewer (DeepView) [43]. Core size and RMSD comparisons on both these reference sets appear in Table 4.

**Table 4:**
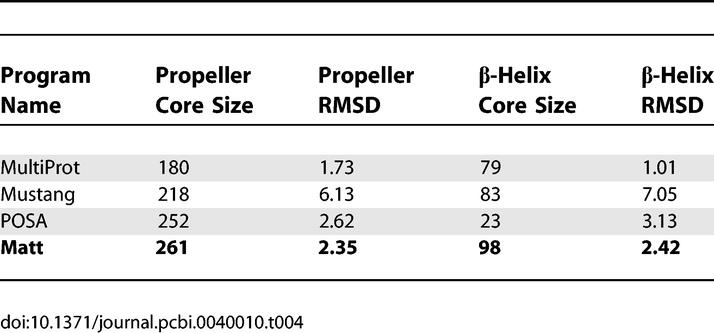
Example of Multiple Structure Alignments in the Two Figures from the SABmark Benchmark Dataset

We then turn to the discrimination problem. Matt, FlexProt, Mustang, and MultiProt were tested on the SABmark superfamily and SABmark twilight zone decoy test suites described in the previous section. Using a method similar to what Gerstein and Levitt [44] did to systematically assess structure alignment programs against a gold standard, length of alignment versus RMSD for the true positives and true negatives were plotted in the plane for all programs. Figure 6 displays the results on the SABmark superfamily set versus SABmark decoys. The separating line marks where the true positive and true negative percentages are roughly equal.

**Figure 6:**
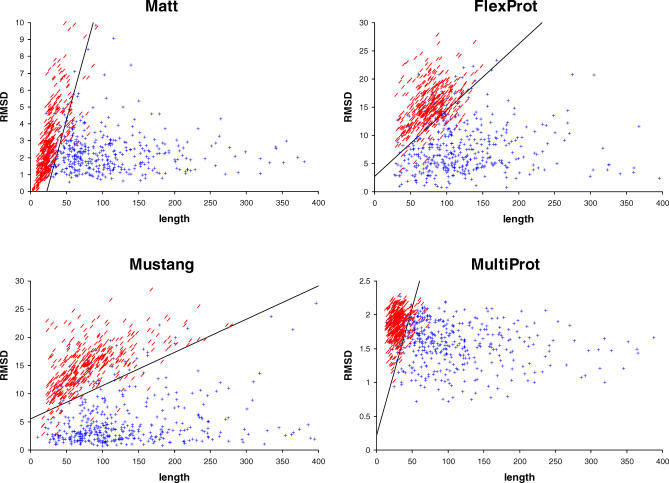
Distinguishing Alignable Structures from Decoys Positive (blue) and SABmark decoy (red) pairwise alignments plotted by RMSD versus number of residues for Matt, FlexProt, MultiProt, and Mustang on the SABmark superfamily set.

When comparing ROC curves over the four different programs, we find that Matt consistently dominates both FlexProt and MultiProt at almost every fixed true positive rate. Mustang does as well. Interestingly, Matt and Mustang are incomparable—on the Superfamily sets, Matt does better than Mustang when the true positive rate is fixed over 93% (90% for the random decoy set), and Mustang does better thereafter. For the twilight zone set, the situation is reversed: SABmark does better than Matt when the true positive rate is between 93% and 98%, but Matt does better between 70% and 92% true positives; then, performance reverses, and Mustang does better below 70% true positive rates. Sample percentages for the four programs near the line where the true positive and true negative percentages are roughly equal appear in Tables 5 and 6 on the superfamily and twilight zone family sets, respectively.

**Table 5:**
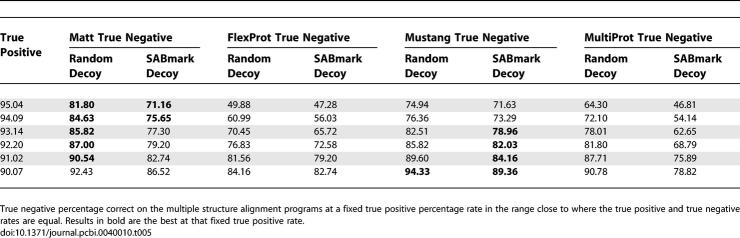
Discrimination Performance on the SABmark Superfamily Set

**Table 6:**
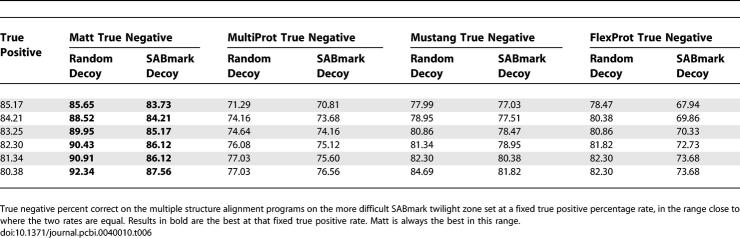
Discrimination Performance on the SABmark Twilight Zone Set

Unsurprisingly, for all four programs, the SABmark decoy set was more difficult to classify than the random decoy set. What was more surprising is how competitive Mustang is with Matt on the discrimination tasks—it is surprising because Mustang was uniformly worse at the alignment problem. In essence, Mustang produces alignments with very high RMSD, but consistently even higher RMSD on the decoy sets. We hypothesize that this may be due to Mustang's use of contact maps, a global measure of fold-fit that may be harder for decoys to match, whereas the decoys may have long regions of local similarity. Matt and Mustang both do qualitatively better at all discrimination tasks than either MultiProt or FlexProt.

Note that in Figure 6 and in both Tables 5 and 6 we have used the RMSD of the best rigid-body transformation that matches the *bent* Matt or FlexProt alignment. At first, we hypothesized that the bent RMSD values might give better discrimination; after all, the bent structures are the local pieces that align really well. However, giving credit for the lower bent RMSD values also greatly improved the RMSD values for the decoy structures, leading in every case to worse performance on the discrimination tasks. Thus, reporting an RMSD value for the rigid superimposition that minimized the RMSD of the residues placed into a bent alignment produced the best separation of true alignments from decoys.

### *p*-Value Calculation

*p*-values for pairwise Matt alignments are calculated based on our 2-D alignment versus RMSD graphs. To calculate *p*-values, we find the slope *m* of a line in the alignment length versus RMSD graph that maximizes the number of elements from the random negative set on one side and has at least 90% of the SABmark positives on the other. This value was chosen because it allows roughly the same percentage of the negative set to be on the other side. These were then used to calculate *z*-scores of the distribution of RMSD —*m* × *length* of the random negative set. These are fit to a standard Gaussian distribution to calculate *p*-values for pairwise alignments.

## Discussion

We introduced the program Matt, which showed that detouring through local flexibility in an AFP alignment algorithm could aid in protein multiple structure alignment. We suggest that looking at local bends in protein structure captures similarity well for fundamental biological as well as for mathematical reasons. In particular, Matt may be both capturing flexibility inherent naturally in some protein structures themselves and modeling structural distortions in the common core that arise as proteins become more evolutionarily distant.

Matt runs in *O*(*k* ^2^ *n* ^3^ log *n*) time, where *k* is the number of sequences participating in the alignment and *n* is the length of the longest sequence in the alignment. Compared with Mustang and MultiProt, Matt takes about the same amount of time to complete running on the benchmark datasets. We find that Matt is typically several times slower on most sets of structures; however, there are a small percentage of sets of substructures in both benchmark datasets where both Mustang and MultiProt take orders of magnitude more time to complete their alignments than they do on the typical set of structures. Matt is saved in these difficult cases with lots of self-similar substructures by its implementation of the oct-trees, which allows for better pruning of the search space. Matt also supports a multithreaded implementation, which is faster still. Matt is sufficiently fast that it should be feasible to construct a set of reference alignments for similar fold classes based on Matt, covering the space of all known folds, which we intend to do.

Examining some of the Matt alignments by hand, the program seems to typically do a better job of aligning the ends of α-helices and β-strands than its competitors; we therefore suggest that Matt may be a useful tool for construction of better sequence profiles for protein structure prediction, and for the construction of better templates for protein threading libraries. For these applications, both Matt's rigid unbent alignments and the geometrically impossible bent alignments may be of interest.

Finally, looking at how Matt (or the other structure aligners) performs on the two SABmark benchmarks raises again the philosophical question of how far into the twilight zone it makes sense to align protein structures. Clearly at the superfamily level, there is typically substantial structural similarity. At the twilight zone level, there is much increased divergence. Examining these alignments in more details may help develop our intuition about the limits of comparative modeling for protein structure prediction as we go further into the twilight zone.

## Methods

### Pairwise alignment.

First, it is useful consider Matt's behavior on pairwise alignments. In what follows, we assume structures are sequentially ordered from their N terminal to their C terminal.

*Notation and definitions.* A block denotes the C-alpha atoms of a set of five to nine adjacent amino acid residues in a protein structure. For block *B*, let *b_h_* denote the first residue and *b_t_* denote the last residue of the block. A block pair is a pair of blocks of equal length, one from each of a pair of structures. For block pair *BC*, define *T_CB_* to be the minimum RMSD transformation that is applied to the second structure to align its C-alpha carbons against the first, and let *RMS_T_* denote the RMSD of the two blocks under *T*. Minimum transformations are calculated using the standard classical singular value decomposition method of Kabsch [26]. For block pair *BC*,

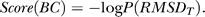
Here, *p*-values are estimated by generating a table of random five-residue RMSD alignments of the NCBI nonredundant PDB [45] (update dated 7 May 2007) with a BLAST E-value cutoff of 10^−7^. For longer block pairs, negative log *p*-values for a given RMSD are assumed to increase linearly with respect to alignment length. (This assumption was verified empirically to be approximately true in the relevant range.) Let *S* be a set of block pairs. Two block pairs *BC* and *DE* are sequential if *b_t_* precedes *d_h_* (and *c_t_* precedes *e_h_*) and there is no block pair with any residue that lies between them in *S*. A set of block pairs is called an *assembly* if it consists of block pairs *P*_1_, *P*_2_ *, …, P_n_* such that *P_i_ and P_i_*_+1_ are sequential. Note that the definition of sequential means that the *P_i_* will be non-overlapping and seen in both structures in precisely this order; see Figure 7.

**Figure 7:**
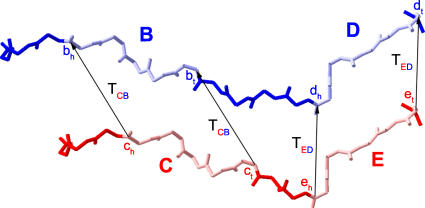
Two Sequential Block Pairs that Could Form Part of an Assembly Block pair *BC* precedes block pair *DE* because *B* precedes *D* and *C* precedes *E* in their respective protein sequences.

### Matt assembly.

A Matt pass uses dynamic programming to create an assembly of block pairs. A pass takes an assembly and three cutoff values as input and outputs a new assembly. The new assembly contains all the block pairs of the original assembly. The three cutoffs are a maximum block-pair RMSD cutoff and maximum values for the displacement and relative angles for each sequential pair of blocks (the “translations” and “twists,” respectively, of Matt's acronym). Using multiple passes and relaxing cutoffs in each successive pass results in favoring strongly conserved regions while still detecting more weakly conserved regions.

All Matt results in this paper use a three-pass algorithm. All three passes use a cutoff of 45° on the angle between the transformations of two sequential block pairs. The first pass is restricted to block pairs with a minimum negative log *p*-value of 2.0, and sequential block pairs must have a displacement no greater than 4. The second pass uses a minimum negative log *p*-value of 1.6 and a maximum displacement of sequential pairs of 5. The last pass uses cutoffs of 0.6 and 10, respectively. The values of these cutoffs were determined by training on a random 20% of the Homstrad benchmark dataset.

More formally, the score of a set of block pairs *S* is the sum of the alignment scores of the individual block pairs and a bonus based on how consistent the transformations associated with the two block pairs are. This score is used internally throughout the construction of the alignment, but is not used to determine the *p*-value, which is instead computed directly based on length and RMSD of the resulting alignments; see the section on calculating *p*-values at the end of the Introduction.

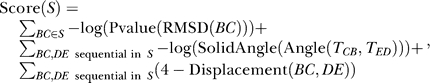
where

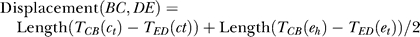
and Length is defined to be ordinary Euclidean distance, Angle is the angle of *T_CB_T_ED_* ^−1^ in axis-angle form, and SolidAngle is the solid angle associated with an angle, measured as a fraction of the sphere. SolidAngle(*θ*) ranges from 0 to 1 and is the probability of a given ray and a random point in 3-D space forming an angle less than *θ*. To prevent overflow, if Angle has a value below 0.1 radians, it is set to 0.1 radians.

We note that a naive implementation of dynamic programming would use *O*(*n* ^4^) time (where *n* is the length of the longer string), as there are *O*(*n* ^2^) block pairs. We reduce this to an *O*(*n* ^3^ log *n*) algorithm by incorporating geometric knowledge of the structural transformations to reduce the search space. In particular, for each of structure two's C-alpha atoms, an oct-tree [46] that partitions Euclidean space based on the transformed positions of that C-alpha atom is created. Each transformation comes from a previously considered block pair that ends with the C-alpha atom in question. When the dynamic program is assembling a block pair starting with the *k*th C-alpha atom in structure two, it searches the oct-trees for all of structure two's C-alpha atoms before *k* for compatible transformations (meaning the associated transformations of the atom are within the distance cutoff).

### Final output.

The final output is an assembly *A*, a bent assembly *A*′ (only output with a command-line option), BentRMSD(*A*′), UnbentRMSD(*A*), and a *p*-value for the assembly.

The result of the dynamic programming algorithm described above gives the bent alignment *A*′, together with a set of local transformations (allowing translations and rotations) that produced the alignment. Its associated RMSD is BentRMSD(*A*′).

To produce the unbent alignment, the set of which residues are to be placed into alignment is retained from *A*′, and all geometric information is discarded. Then, the best global rigid-body transformation that minimizes the RMSD of this alignment is found using the classical SVD method of Kabsch [26]. The result is the *rigid-bent* RMSD, which is not output explicitly, but implicitly forms the basis for the *p*-value calculation (as described in the *p*-value section at the end of the Introduction). Note that so far the sequence alignment from the bent step is unchanged, and thus only includes sets of contiguous residues that are at least 5 Å in length. A final pass then greedily adds back shorter segments of four or fewer residues that fall between already aligned fragments but whose RMSD under the global transformation falls below a user-settable cutoff. The resulting alignment is *A*, with its associated *unbent RMSD*(*A*).

### Multiple alignment.

In this subsection, we extend the definitions of assemblies to sets of more than two structures in the natural way. The input to each iteration of the Matt multiple alignment algorithm is the set of structures to be aligned that have been partitioned into sets, where the structures in each set are multiply aligned in an assembly. The output of the iteration leaves all but two sets unchanged; the structures in those two sets are merged into a single set with a new multiple alignment assembly. (The initial iteration of Matt places each structure into its own set and is equivalent to the pairwise alignment algorithm described above.)

Analogous to blocks, we define *block-tuples*. A *block-tuple* is an aligned set of already aligned equal-length blocks. A block-tuple pair is a set of two block-tuples, one from each of two sets of aligned structures.

At each iteration, for every pair of sets in the partition, Matt uses the merge procedure detailed below to combine the two assemblies into a single assembly and computes its score. It then chooses the best-scoring pair of sets to merge in that iteration. Matt terminates when all structures have been aligned into a single assembly, outputting the assembly, bent RMSD, and unbent RMSD.

*Merging.* Here is a high-level description of the steps of the merge procedure. The merge procedure take as input two assemblies, each possibly containing multiple structures. Each step is explained in more detail. (1) Find the pair of structures, *a*_0_ from assembly *A* and *b*_0_ from assembly *B*, that have the highest-scoring pairwise structural alignment, according to the pairwise structural alignment algorithm of the previous section. (2) For each block *β* of *a*_0_, let *T_β_* denote the transformation of *β* back to its original atomic coordinates. Transform every block in the block-tuple associated with *β* in *A* by *T_β_*. Do the same for each block of *b*_0_ and its associated block-tuple. (3) Within each *A* and *B* separately, realign all blocks within each block-tuple to reduce RMSD using the LocalOpt procedure (described below). (4) Still within each *A* and *B* separately, an extension step is performed than can lengthen block-tuples into unaligned regions or other block-tuples. Note that this step may temporarily produce overlapping blocks, but that this is corrected later, and only non-overlapping block-tuples are returned at the end of the merge procedure. (5) Taking the set of all aligned block-tuples of *A*, we consider all sets of five to nine adjacent residues entirely contained within one of these block-tuples by sliding a window of appropriate length. We do the same for the block-tuples of *B*. These become the building blocks of new block-tuple pairs in the merged assembly. Just as in the pairwise case, all equal-length building blocks, one each from *A* and *B*, are pairwise aligned and scored. Note that the RMSD algorithm easily generalizes to align sets of aligned residues by treating each aligned *k*-length set of *m* structures against *n* structures as a single RMSD alignment of *kmn* residues. Let *T_BA_*(*r*,*s*) be the transformation that produces the lowest RMSD on the block-tuple pair *r* ∈ *A*, *s* ∈ *B*. (6) Dynamic programming is used to find the optimal assembly of pairs of new block-tuples. The dynamic programming algorithm in the multiple structural case has the same form as in the pairwise case. The angle penalty is calculated as before from *T_BA_*(*r*,*s*). The displacement penalty is calculated slightly differently. In each residue position, an average C-alpha atom position is calculated for both *A* and *B*. The displacement is calculated using the averages as if they were positions of atoms of single structures in a pairwise alignment. Note that as in the pairwise case, this algorithm does not allow block-tuples to overlap, so the final set of block-tuples that are aligned by the merge procedure will be a legal assembly.

The merge procedure has now been specified except for LocalOpt and the extension procedure. We now explain both these steps in detail.

*LocalOpt.* LocalOpt acts independently on *A* and *B*; therefore, it is described here only on *A*. The intuition for the LocalOpt step comes from merges in the previous iterations. The block-tuple alignment that an assembly inherits is the result of a global RMSD alignment of the block-tuple of potentially many protein structures. Therefore, while keeping the residue alignments fixed, it is often still possible to transform the coordinates of the C-alpha atoms to improve bent RMSD. LocalOpt acts separately on each block-tuple in the assembly. In particular, if *a*_0_, *a*_1_, …, *a_r_* are the structures within a block-tuple (where *a*_0_ is the reference structure as defined by merge), each *a_i_* is removed in turn and then realigned to minimize RMSD to the other *r* structures for each block-tuple.

*Extension phase.* In the extension phase, both ends of block-tuples of five to nine residues are explored in order to determine if additional adjacent residues have a good multiple alignment. The LocalOpt transformations associated with each block-tuple in each structure are applied to the residues immediately before and after the block-tuple. If the average distance between all pairs of residues in all structures before or after a block-tuple is less than 5 Å, the residues are added to the block-tuple. This is done in a greedy fashion. Note that this allows block-tuples to be longer than nine residues or even to overlap (though in practice, we do not allow them to overlap by any more than five residues to bound computational time).

*Final output.* When only one assembly remains, as in the pairwise case, this produces the bent alignment. As before, a final realignment and extension phase is done in which additional *i*-residue segments are greedily added to the common core, for *i* = 4 down to 1, provided their average RMSD lies below a (user-settable) cutoff. This cutoff is solely responsible for the size of common core/RMSD tradeoff found in Figures 2 and 3. All residues that were aligned in the iterative phase are kept in the final alignments. Block-tuples are not allowed to overlap in the final extension phase. A new RMSD alignment of the original unbent structures is done, aligning residues according to the extended block-tuples. This assembly, its RMSD, and the sets of aligned residues are the algorithm's unbent output. A *p*-value is not output when a group of more than two structures is to be aligned.

## Abbreviations

AFP: aligned fragment pair
RMSD: root mean squared deviation

## References

[pcbi-0040010-b001] Levitt M, Gerstein M (1998). A unified statistical framework for sequence comparison and structure comparison. Proc Natl Acad Sci U S A.

[pcbi-0040010-b002] Chothia C, Lesk A (1986). The relation between sequence and structure in proteins. EMBO J.

[pcbi-0040010-b003] Rost B (1999). Twilight zone of protein sequence alignments. Protein Eng.

[pcbi-0040010-b004] Dunbrack RL (2006). Sequence comparison and protein structure prediction. Curr Opin Struct Biol.

[pcbi-0040010-b005] Abagyan RA, Batalov S (1997). Do aligned sequences share the same fold?. J Mol Biol.

[pcbi-0040010-b006] Edgar R, Batzoglou S (2006). Multiple sequence alignment. Curr Opin Struct Bio.

[pcbi-0040010-b007] Whisstock JC, Lesk AM (2003). Prediction of protein function from protein sequence and structure. Q Res Biophys.

[pcbi-0040010-b008] Kinch LN, Grishin NV (2002). Evolution of protein structures and functions. Curr Opin Struct Biol.

[pcbi-0040010-b009] Grishin NV (2001). Fold change in evolution of protein structures. J Struct Biol.

[pcbi-0040010-b010] Irving JA, Whisstock JC, Lesk AM (2001). Protein structural alignments and functional genomics. Proteins.

[pcbi-0040010-b011] Panchenko A, Marchler-Bauer A, Bryant SH (1999). Threading with explicit models for evolutionary conservation of structure and sequence. Proteins.

[pcbi-0040010-b012] O'Sullivan O, Suhre K, Abergel C, Higgins D, Notredame C (2004). 3DCoffee: Combining protein sequences and structures within multiple sequence alignments. J Mol Biol.

[pcbi-0040010-b013] Shindyalov I, Bourne P (1998). Protein structure alignment by incremental combinatorial extension (CE) of the optimal path. Protein Eng.

[pcbi-0040010-b014] Ye Y, Godzik A (2003). Flexible structure alignment by chaining aligned fragment pairs allowing twists. Bioinformatics.

[pcbi-0040010-b015] Holm L, Park J (2000). DaliLite workbench for protein structure comparison. Bioinformatics.

[pcbi-0040010-b016] Nussinov R, Wolfson H (1991). Efficient detection of three-dimensional structural motifs in biological macromolecules by computer vision techniques. Proc Natl Acad Sci U S A.

[pcbi-0040010-b017] Dror O, Benyamini H, Nussinov R, Wolfson H (2003). MASS: Multiple structural alignment by secondary structures. Bioinformatics.

[pcbi-0040010-b018] Dror O, Benyamini H, Nussinov R, Wolfson H (2003). Multiple structure alignment by secondary structures: Algorithm and applications. Protein Sci.

[pcbi-0040010-b019] Kolbeck B, May P, Schmidt-Goenner T, Steinke T, Knapp1 EW (2006). Connectivity independent protein-structure alignment: A hierarchical approach. BMC Bioinformatics.

[pcbi-0040010-b020] Xu J, Jiao F, Berger B (2007). A parameterized algorithm for protein structure alignment. J Comput Biol.

[pcbi-0040010-b021] Yuan X, Bystroff C (2005). Non-sequential structure-based alignments reveal topology-independent core packing arrangements in proteins. Bioinformatics.

[pcbi-0040010-b022] Goldman D, Istrail S, Papadimitriou CH, Beame P (1999). Algorithmic aspects of protein structure similarity. Proceedings of the 40th Annual Symposium on Foundations of Computer Science.

[pcbi-0040010-b023] Wang L, Jiang T (1994). On the complexity of multiple sequence alignment. J Comput Biol.

[pcbi-0040010-b024] Kolodny R, Linial N (2004). Approximate protein structural alignment in polynomial time. Proc Natl Acad Sci U S A.

[pcbi-0040010-b025] Eidhammer I, Jonassen I, Taylor WR (2000). Structure comparison and structure patterns. J Comput Biol.

[pcbi-0040010-b026] Kabsh W (1978). A discussion of the solution for the best rotation to relate two sets of vectors. Acta Crystallogr A.

[pcbi-0040010-b027] Ye Y, Godzik A (2005). Multiple flexible structure alignment using partial order graphs. Bioinformatics.

[pcbi-0040010-b028] Kolodny R, Koehl P, Levitt M (2005). Comprehensive evaluation of protein structure alignment methods: Scoring by geometric measures. J Mol Biol.

[pcbi-0040010-b029] Jung J, Lee B (2000). Protein structure alignment using environmental profiles. Protein Eng.

[pcbi-0040010-b030] Suyama M, Matsuo Y, Nishikawa K (1997). Comparison of protein structures using 3D profile alignment. J Mol Evol.

[pcbi-0040010-b031] Bonvin A (2006). Flexible protein-protein docking. Curr Opin Struct Biol.

[pcbi-0040010-b032] Echols N, Milburn D, Gerstein M (2003). MolMovDB: Analysis and visualization of conformational change and structural flexibility. Nucleic Acids Res.

[pcbi-0040010-b033] Lemmen C, Lengauer T, Klebe G (1998). FlexS: A method for fast flexible ligand superposition. J Medicinal Chem.

[pcbi-0040010-b034] Schueler-Furman O, Wang C, Bradley P, Misura K, Baker D (2005). Review: Progress in modeling of protein structures and interactions. Science.

[pcbi-0040010-b035] Singh R, Berger B, Altman R, Jung T, Klein T, Dunker K, Hunter L (2005). ChainTweak: Sampling from the neighbourhood of a protein conformation. Proceedings of the 2005 Pacific Symposium on Biocomputing.

[pcbi-0040010-b036] Shatsky M, Nussinov R, Wolfson H (2002). Flexible protein alignment and hinge detection. Proteins.

[pcbi-0040010-b037] Mizuguchi K, Deane C, Blundell TL, Overington J (1998). HOMSTRAD: A database of protein structure alignments for homologous families. Protein Sci.

[pcbi-0040010-b038] VanWalle I, Lasters I, Wyns L (2005). SABmark—A benchmark for sequence alignment that covers the entire known fold space. Bioinformatics.

[pcbi-0040010-b039] Barton G, Sternberg M (1987). A strategy for the rapid multiple alignment of protein sequences: Confidence levels from tertiary structure comparisons. J Mol Biol.

[pcbi-0040010-b040] Murzin A, Brenner S, Hubbard T, Chothia C (1995). SCOP: A structural classification of proteins database for the investigation of sequences and structures. J Mol Biol.

[pcbi-0040010-b041] Shatsky M, Nussinov R, Wolfson H (2004). A method for simultaneous alignment of multiple protein structures. Proteins.

[pcbi-0040010-b042] Konagurthu A, Whisstock J, Stuckey P, Lesk A (2006). MUSTANG: A multiple structural alignment algorithm. Proteins.

[pcbi-0040010-b043] Guex N, Peitsch M (1997). SWISS-MODEL and the Swiss-PdbViewer: An environment for comparative protein modeling. Electrophoresis.

[pcbi-0040010-b044] Gerstein M, Levitt M (1998). Comprehensive assessment of automatic structural alignment against a manual standard, the SCOP classification of proteins. Prot Sci.

[pcbi-0040010-b045] Hobohm U, Sander C (1994). Enlarged representative set of protein structures. Protein Science.

[pcbi-0040010-b046] Jackins C, Tanimoto S (1983). Quad-trees, Oct-trees, and K-trees: A generalized approach to recursive decomposition of euclidean space. IEEE Trans Pattern Anal Mach Intell.

